# Expression of selected genes isolated from whole blood, liver and obex in lambs with experimental classical scrapie and healthy controls, showing a systemic innate immune response at the clinical end-stage

**DOI:** 10.1186/s12917-018-1607-9

**Published:** 2018-09-12

**Authors:** Siv Meling, Kerstin Skovgaard, Kjetil Bårdsen, Peter Mikael Helweg Heegaard, Martha J. Ulvund

**Affiliations:** 1 Faculty of Veterinary Medicine, Norwegian University of Life Sciences, Sandnes, Norway; 2 Department of Biotechnology and Biomedicine, Technical University of Denmark, Kemitorvet, 2800 Lyngby, Denmark

**Keywords:** Classical scrapie, Innate immune response, qPCR, Whole blood, Liver tissue, Brain tissue, Sheep

## Abstract

**Background:**

Incubation period, disease progression, pathology and clinical presentation of classical scrapie in sheep are highly dependent on *PRNP* genotype, time and route of inoculation and prion strain. Our experimental model with pre-colostrum inoculation of homozygous VRQ lambs has shown to be an effective model with extensive PrP^Sc^ dissemination in lymphatic tissue and a short incubation period with severe clinical disease. Serum protein analysis has shown an elevation of acute phase proteins in the clinical stages of this experimental model, and here, we investigate changes in gene expression in whole blood, liver and brain.

**Results:**

The animals in the scrapie group showed severe signs of illness 22 weeks post inoculation necessitating euthanasia at 23 weeks post inoculation. This severe clinical presentation was accompanied by changes in expression of several genes. The following genes were differentially expressed in whole blood: *TLR2, TLR4, C3, IL1B, LF* and *SAA,* in liver tissue, the following genes differentially expressed: *TNF-α, SAA, HP, CP, AAT, TTR* and *TF,* and in the brain tissue, the following genes were differentially expressed: *HP, CP, ALB* and *TTR.*

**Conclusions:**

We report a strong and evident transcriptional innate immune response in the terminal stage of classical scrapie in these animals. The *PRNP* genotype and time of inoculation are believed to contribute to the clinical presentation, including the extensive dissemination of PrP^Sc^ throughout the lymphatic tissue.

**Electronic supplementary material:**

The online version of this article (10.1186/s12917-018-1607-9) contains supplementary material, which is available to authorized users.

## Background

Prion diseases are a group of diseases with common pathology of the central nervous system including neuronal degeneration, vacuolation and gliosis [1]. The causative agent is an infectious protein, called the prion (PrP^Sc^), which is an abnormal isoform of the cellular prion protein, PrP^C^, found in many cell types in the body [2]. Prion diseases are also named Transmissible Spongiform Encephalopathies (TSEs), and scrapie is the TSE that affects sheep and goats. Sheep can become infected early in life, around and soon after birth, from infectious prions in foetal fluids and membranes and bodily secretions like colostrum/milk and faeces [3–6].

Susceptibility to classical scrapie in sheep is highly dependent on polymorphism at codons 136, 154 and 171 of the PrP gene (*PRNP*). The five possible alleles, ARR, AHQ, ARH, ARQ and VRQ, give rise to 15 combinations found in sheep, and these are also linked to the different presentations of classical scrapie [7]. These genotypes differ widely in their susceptibility to scrapie, from extreme susceptibility in homozygous V_136_R_154_Q_171_ genotype, to very low susceptibility in homozygous A_136_R_154_R_171_ [8–10]. The PrP genotype not only influences the degree of susceptibility, but also the course of disease, incubation period and clinical picture [7, 11]. This genetic susceptibility is the basis behind the breeding for resistance program successfully implemented in the EU as per EC Regulation 999/2001, where only ewes carrying at least one ARR allele and no VRQ allele, and rams of ARR/ARR genotype, are bred from [12]. Several member states have implemented breeding programs to select for resistance to TSEs in their ovine population, resulting in a marked increase in ARR frequency and a decreased VRQ frequency. There are now reports of a shift in *PRNP* genotypes in national flocks after years of breeding from low susceptibility genotypes [13–16]. Thus, the prevalence of classical scrapie at population level decreased as highly susceptible genotypes are eliminated.

Presentation and progression of disease are influenced by *PRNP* genotype, PrP^Sc^ strain, and route of inoculation. Lateral transmission via the oral route is the most important and commonest route of infection, and in a flock where scrapie is endemic, new-born lambs become infected at birth from their infected dam and/or from the contaminated environment [17]. After oral infection, prions seem to be taken up across the gastrointestinal mucosa by M-cells, and dendritic cells (DCs) and possibly macrophages are involved in the spread to the lymphoreticular system (LRS). Follicular dendritic cells (FDCs) within the LRS, are the site of prion replication, but the extent and speed of lymphatic involvement is dependent on *PRNP* genotype [18]. Sheep carrying the least susceptible ARR allele show minimal PrP^Sc^ deposition within the LRS, while carriers of the VRQ allele are associated with extensive PrP^Sc^ deposition [19–24]. When lambs with the most susceptible genotype are orally challenged with scrapie infection, PrP^Sc^ is detected very early in lymphoid tissues associated with the gastrointestinal tract, and is followed by dissemination of PrP^Sc^ to other, non-gastrointestinal, lymphoid tissues [24–26]. Due to this early PrP^Sc^ accumulation in lymphatic tissue in certain genotypes, the European Food Safety Authority considers young stock, less than six months of age and from TSE infected flocks, as potentially highly infectious [27].

The pathogenesis and progression of TSE in different species is still not fully understood, and sheep with classical scrapie is a good model to study the disease mechanisms. Pre-colostrum inoculation of lambs at birth is an effective model that has shown to produce severe clinical disease after a relatively short incubation period [28]. One can say, our model produces a “worse-case scenario” of prion disease, where the genotype, donor material and time of infection are optimal. Ersdal and co-workers reported a study of PrP^Sc^ uptake and dissemination in an earlier experimental model, where older lambs where orally inoculated, with the same VRQ-prion donor material, between 46 and 61 days of age [22]. Later, this experimental model was repeated, only this time the lambs where inoculated at birth, and produced a much shorter incubation period and more severe clinical disease [28]. Both these models revealed an early and strong dissemination of PrP^Sc^ in lymphatic tissue in combination with relatively moderate cytopathogenic changes in the CNS in VRQ/VRQ animals. We have earlier published our findings of an acute phase response in lambs inoculated at birth, evident at serum protein level [29]. In the present study, transcriptional changes of the above lambs were (further) studied. This article describes the expression of a selection of genes in RNA isolated from whole blood, liver and posterior obex of healthy and scrapie affected lambs.

Many genes are expressed by leukocytes in the blood as a systemic response to pathology in peripheral tissues. Injured and diseased tissues and organs will alter the gene expression profile in blood leukocytes and in the liver, which is the main producer of acute phase proteins associated with an innate immune response. Several studies of gene expression in circulating blood cells have revealed expression profiles characteristic for a wide variety of diseases [30–32]. Gene expression studies of whole blood can thus improve our knowledge of disease related processes regardless of which tissue is primarily affected. White blood cells have an important role in the immune system, and, although scrapie is known to not produce a specific immune response per se, cells of the monocyte line seem to play an important role in the uptake from the gut and dissemination of PrP^Sc^ to lymphoid organs and further into the CNS resulting in gliosis and astrocytosis [20, 22].

Collection and stabilization of total RNA from whole blood using PAXgene Blood RNA Tubes (BD Biosciences) offer a robust and simple method that minimize sampling-to-sampling differences. PAXgene technology capture the RNA profiles at the time of collection of all cell types in whole blood, including peripheral blood monocytes, lymphocytes, erythrocytes/reticulocytes, granulocytes and platelets [33].

The aim of this study was to investigate how scrapie infection influences the gene expression profile of selected genes in whole blood during the preclinical incubation period and through to the terminal clinical end stage. Further, the gene expression patterns were studied in liver and posterior obex at the terminal end stage.

## Methods

### Animals

The animals and experimental model used in this project were described in detail previously, and all animal procedures were approved by the Norwegian Animal Research Authority [28, 29]. Briefly, nine lambs were inoculated by stomach tube, at birth and before colostrum intake, with pooled brain material from either healthy, scrapie free sheep (control group) or from confirmed cases of classical scrapie (scrapie group). All the lambs originated from the commercial sheep flock belonging to the Norwegian University of Life Sciences, Faculty of Veterinary Medicine, Department of Production Animal Clinical Sciences, Section of Small Ruminant Research, 4325 Sandnes, Norway. Within this flock, a number of animals have selectively been bred to maintain the homozygous V_136_R_154_Q_171_ *PRNP* genotype. The control group consisted of two twins (four lambs) and the scrapie group consisted of triplets and twins (five lambs). The lambs and their dams were housed individually until euthanasia at 23 weeks of age. All the animals were inspected daily, and the scrapie group was under video surveillance. Euthanasia was performed by an intravenous injection of sodium pentobarbital, using the jugular vein.

### Blood and tissue collection and RNA isolation

Blood samples were collected at regular intervals from the jugular vein into appropriate blood tubes, depending on the requirement for the different analyses. Whole blood (2.5 mL) was collected at 12, 14, 16, 20, 22 and 23 weeks post inoculation (wpi) in PAXgene Blood RNA Tubes (Qiagen/BD Company). All the samples were handled according to manufacturer’s instructions and stored at minus 70 °C until analysis.

Brain and liver tissues were removed immediately post mortem and placed in RNA*later* (Qiagen) prior to storage at minus 70 °C. Total RNA was extracted and purified from stabilized blood samples manually, using PAXgene® Blood miRNA Kit (Qiagen/BD Company) according to manufacturer’s instructions using the protocol for manual purification. The RNA eluate was immediately stored at minus70°C.

Total RNA was isolated from 20 to 25 mg tissue sections, lysed and homogenized, from the posterior obex region in the brain and from the liver using RNeasy® Lipid Tissue Midi Kit and (Qiagen) and RNeasy® Lipid Tissue Mini Kit (Qiagen), respectively, including digestion of DNA using RNase-Free DNase kit (Qiagen).

Total RNA was quantified by UV spectrometry at 260 nm using a NanoDrop ND-1000 spectrophotometer (Saveen and Werner AB, Limhamn, Sweden). In addition, for each sample 260/280 and 260/230 ratios were provided, enabling RNA sample purity estimations. RNA integrity was evaluated using the Agilent® 2100 Bioanalyzer with Agilent RNA 6000 Nano Kit (Agilent Technologies, Waldbronn, Germany), according to manufacturer’s instructions. Each total RNA sample was assigned an RNA Integrity Number (RIN) ranging from 1 to 10. The mean and standard deviation of all the RIN values were calculated and are shown in Additional file 1.

### Target genes

Two different primer pairs were designed to amplify different regions of each gene to ensure high quality results. Sequences used for primer design were obtained from public database (GenBank, NCBI) and are listed together with reaction conditions in Additional file 2. Primers were designed using Primer3 (http://bioinfo.ut.ee/primer3-0.4.0/primer3/) and synthesized at TAG Copenhagen A/S (Copenhagen, Denmark). BLAST searches were performed to confirm gene specificity of the primer sequence, and to show the absence of polymorphisms at the primer site. Whenever possible, only sequences from *Ovis aries* were used, if not, sequences specific for *Bos taurus* or *Capra ibex* were used. Further, NetPrimer software available at http://www.premierbiosoft.com was used to obtain a rating value for each primer pair.

### High-throughput qPCR

Two separate technical replicates of complementary DNA (cDNA) were synthesised from each RNA sample using 300 ng of total RNA using the QuantiTect reverse-transcription kit (Qiagen®/BD Company), according to the manufacturer’s instructions. All samples were diluted, with a dilution factor of 0.13, in low EDTA TE-buffer (VWR, APLIA8569.0500) in a total solution of 20 μl.

Custom-designed primers (TAG Copenhagen, Copenhagen, Denmark) were diluted to 100 μM. For preamplification and qPCR, each primer pair, consisting of forward and reverse primers was further diluted in low EDTA TE-buffer (VWR, APLIA8569.0500) to reach a final concentration of 20 μM. Then a primer mix was created from 5 μl of each 20 μM primer pair and low EDTA TE-buffer in a 500 μl, 200 nM solution. A PreAmp mix, consisting of 5 μl TaqMan PreAmp Master Mix (Applied Biosystems, PN 4391128) and 2.5 μl 200 nM primer mix, was added to 2.5 μl of each cDNA sample. This mix was incubated under thermal cycling conditions as follows; initially held at 95 °C for 10 min, followed by 16 pre-amplification cycles of 15 s at 95 °C and 4 min at 60 °C, before returning to 4 °C. Before storage at − 20 °C, each sample was diluted 1:4 in low EDTA TE-buffer. The liver samples were optimised by a second run, L2; using a 1:10 dilution.

High-throughput qPCR was performed in 48.48 Dynamic Arrays using the BioMark thermocycler (Fluidigm Corporation, CA, USA) following the manufacturer’s protocol. The 48.48 dynamic array enables 2304 separate and simultaneous qPCR reactions from 48 samples and 48 primers in one operation. Each 48.48 dynamic array was prepared with control line fluid and primed in an IFC Controller MX (Fluidigm Corporation, CA, USA) while assays and samples were prepared. The Assay master mix (2.75 μl), consisted of 2.5 μl loading reagent (85,000,800 Sample and Assay Loading kit, Fluidigm) and 0.25 μl low EDTA TE-buffer, were combined with 2.3 μl 20 μM of each specific primer pair before loading each corresponding assay inlets on the 48.48 dynamic array. The Pre-sample mix (4.5 μl), consisting of 3 μl TaqMan Gene Expression Master mix (Applied Biosystems, PN 4369016), 0.3 μl DNA binding dye (100–0388, Fluidigm), 0.3 μl EvaGreen (VWR, BTIU31000) and 0.9 μl low EDTA TE-buffer, and 1.5 μl preamplified cDNA sample were added to each corresponding sample inlets on the 48.48 dynamic array. The 48.48 dynamic array was then returned to the IFC Controller to load samples and assays into the Integrated Fluidic Circuit of the dynamic array. The 48.48 dynamic array was placed in the BioMark HD instrument for qPCR with a ten-minute hot start phase at 95 °C followed by 35 cycles of denaturing at 95 °C for 15 s and annealing/elongation at 60 °C for 1 min. Melting curves were generated after each run to confirm the presence of a single PCR product (from 60 °C to 95 °C, increasing 1 °C/3 s). Non-template controls, non-reverse transcriptase controls and three interplate calibrators were included on each dynamic array if data from multiple dynamic arrays were analysed.

### Data collection and pre-processing

RT-qPCR data was collected using Fluidigm Real-Time PCR Analysis Software Version 3.0.2 (Fluidigm Corporation, CA, USA). Melting curves and non-template control (NTC) were used to monitor non-specific amplification and sample contamination. Non-reverse transcriptase controls were used to assess potential genomic DNA contamination. Each sample was examined to confirm a single PCR product, and lack of such product resulted in the rejection of that quantification cycle (Cq) value.

Expression data were pre-processed and analysed using GenEx Pro Version 5.3.6 (MultiD Analyses AB, Goteborg, Sweden). Pre-processed data were separated into four data subsets (blood, obex, liver1 and liver2) according to type of sample and time of analysis, and data analysis was performed with GenEx Pro Version 5.3.6 in the following order: 1. Initial visual evaluation of melting curves and amplification curves; 2. Efficiency correction. PCR amplification efficiency was established by the means of calibration curves and determination of the slope (S) of the log-linear portion. The calibration curve was constructed from dilution series (4 × 4) using a pool consisting of 1 μl of each sample tested, and diluted into following relative concentrations: F1 0.333, F2 0.067, F3 0.0133 and F4 0.00267. Both slope value (m) and correlation coefficient R^2^ for each assay was determined using the above. Efficiency value (E) was calculated as follows: E = 10^1/m^ – 1, and used for the efficiency correction of each primer assay individually in GenEx. Any assay with E values less than 0.8 or higher than 1.1, and R^2^ values less than 0.95, were discarded and excluded for further analysis; 3. Inter-plate calibration (IPC): based on the three interplate calibrators; 4. Normalization to reference genes. Six different genes (*RPLP0*, *PPIA*, *UBC*, *HPRT1*, *GAPDH* and *SDHA*) were evaluated using GeNorm and NormFinder incorporated in GenEx; 5. Averaging technical cDNA replicates. Before cDNA technical replicates were averaged, the data set was inspected for level of variation between replicates. A maximum of 20% of difference in Cq between the two replicates was accepted.; 6. The relative expression of each sample analysed, was calculated using the mean Cq value of the control croup as a base line. Any sample with higher Cq (lower expression) than mean Cq of control group, get a value below 1, and any sample with lower Cq (higher expression) than mean Cq of control group, get a value above 1. This was done for each primer assay individually; 7. Finally, the data was converted into logarithmic scale, using log_2_ transformation for all statistical analysis. Fold change was calculated by scaling the relative expression data. A scaling factor was calculated based on the average relative expression (fold change) in the control group was scaled to 1. Individual expression data was multiplied by the scaling factor, giving individual fold change. Average fold change and standard error of the mean (SEM) was calculated for each group/primer assay. When relative expression (RE) was below 1, it was translated to a negative fold change value using − 2^-(log^_2_^RE)^, i.e. a relative expression of 0.8, gave a fold change of − 1.2.

### Statistical analysis

The data were analysed separately using statistical features of GenEx software (MultiD). The pre-processed data from the qPCR analyses was separated into subsets of data, based on time and type of sampling. The non-parametric statistical model, Mann-Whitney U Test, was used to calculate *p*-values between scrapie and control groups in each of the data subsets, and significant p-value was set to *p* < 0.05. Due to the small group size, p-values should be interpreted with some caution. Genes were considered as differentially expressed if they showed a fold change of ≥1.5 and reached significant p-values.

## Results

### Animals

**Fig. 1:**
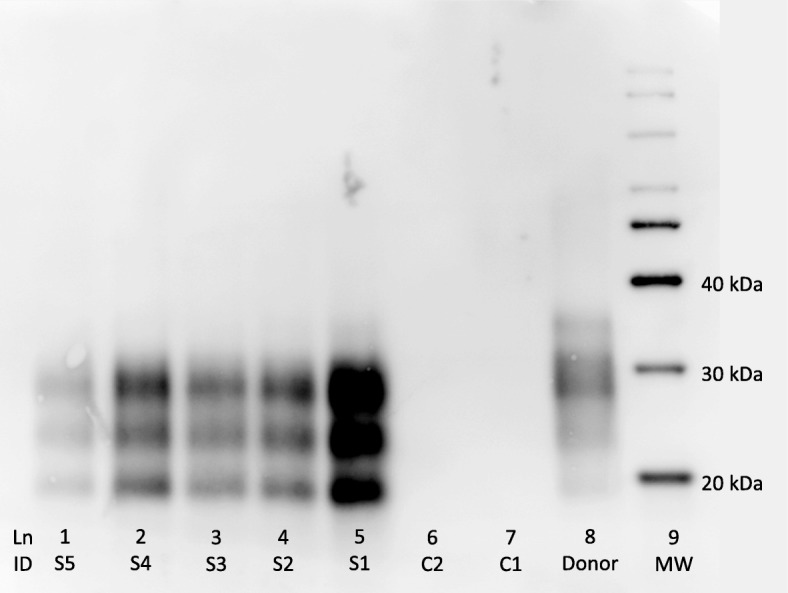
Western Blot image. Western immunoblot using P4 antibody for the detection of PrP^Sc^ in equal amount of homogenated brain tissue from animals and inoculation material used in the experiment (TeSeE Western Blot, Bio-Rad). Lanes 1–5 represent the five animals in the scrapie group, and lanes 6 and 7 represent two animals in the control group. Lane 8 represents inoculation material (donor) used in the scrapie group and a molecular marker was placed in lane 9. PrP^Sc^ was detected in inoculation material and in all the animals from the scrapie group

Animals in the control group remained healthy without any clinical symptoms for the full experimental period. No obvious clinical signs of illness were visible in the scrapie group until 22 wpi, although subtle signs of unspecific ill-thrift were observed in two animals from 19 wpi. By the end of 22 wpi., all the animals in the scrapie group showed signs of disease, such as weakness, dullness and lethargy. Two animals in the infected group displayed an accelerated worsening of clinical signs to terminal recumbency and severe depression. This sudden change, over a few days, made euthanasia necessary. Brain tissue from all the animals in both groups was examined for PrP^Sc^ by immunohistochemistry (IHC) and Western Blot (WB), and only the animals in the scrapie group were found PrP^Sc^-positive (Fig. 1). There were moderate vacuolation of neuropil and neurons with marked distribution of PrP^Sc^ deposits in the CNS, and PrP^Sc^ was found to be widely distributed in lymphoid organs (spleen and retropharyngeal lymph nodes), consistent with previous findings [28].

### Gene expression analysis in blood

Statistically differentially expressed genes, fold change and *p*-value are summarized in Table 1. The magnitude of the fold change at each time points, reflects the gene expression alterations relative to the mean level of the control group scaled to 1. Of the 10 target genes analysed after pre-processing, only six were significantly differently expressed in the scrapie group compared to the control group at any time point. No genes were differentially expressed at 12 nor 14 wpi. *IL1B* was differentially expressed at 16 and 22 wpi, and *TLR2, TLR4, C3* and *SAA* were differentially expressed at 22 and 23 wpi. *LF* was only differentially expressed at 23 wpi. Apart from *IL-1B* which was downregulated at 16 wpi, all the others were upregulated. Expression levels of *TLR9, IL1RN, IL8* and *LR/LAMR1* were not significantly altered at any of the time points. (Table 1).

**Table 1:** Mean fold change and *p*-values of selected genes in whole blood at three time points

Gene function	Gene name		Whole blood					
			16 wpi		22 wpi		23 wpi	
			FC ± SEM	p	FC ± SEM	p	FC ± SEM	p
Pattern Recognition Receptor	*TLR2*	Toll-like receptor 2	1.11 ± 0.04	0.73	1.96 ± 0.28	0.02	1.98 ± 0.54	0.03
	*TLR4*	Toll-like receptor 4	−1.41 ± 0.05	0.41	2.14 ± 0.42	0.02	2.00 ± 0.42	0.03
Complement Component	*C3*	Complement component 3	1.57 ± 0.22	0.19	3.04 ± 0.91	0.02	4.74 ± 2.32	0.02
Interleukin	*IL1B*	Interleukin-1β	−1.71 ± 0.03	0.02	1.66 ± 0.16	0.02	−1.24 ± 0.15	> 0.05
Acute Phase Protein	*SAA*	Serum Amyloid A	1.07 ± 0.35	0.73	5.00 ± 1.49	0.02	21.99 ± 18.03	0.03
	*LF*	Lactoferrin	1.05 ± 0.35	1.00	4.64 ± 2.64	0.06	13.70 ± 10.35	0.02

Mean fold change (FC) with standard error of the mean (SEM) and p-value of differentially expressed genes in whole blood in the scrapie group. The mean fold change value is relative to the mean of the control group which is scaled to 1, at each of the different times (weeks) post inoculation

### Gene expression analysis in liver at 23 wpi

Due to the small group size, only descriptive analyses were performed. Differentially expressed genes of the scrapie group relative to the control group with a fold change of ≥1.5 can be seen in Table 2. The fold change value is relative to the mean expression level in the control group, set as value 1. Of the 12 target genes analysed after pre-processing, seven genes were expressed with fold change ≥1.5 (Table 2). Three genes were upregulated (*SAA, Hp* and *Cp*) and four were downregulated (*TNFa, AAT, TF* and *TTR*).

**Table 2:** Fold change of selected genes in liver tissue

Gene function	Gene Name		Fold change ± SEM
TNF super family	*TNFa*	Tumor Necrosis Factor alfa	−1.77 ± 0.12
Acute phase protein	*SAA*	Serum Amyloid A	89.98 ± 46.32
	*TF*	Transferrin	−1.68 ± 0.10
	*Hp*	Haptoglobin	542.96 ± 216.93
	*Cp*	Ceruloplasmin	1.98 ± 0.46
	*AAT*	Alpha-1 antitrypsin	−2.37 ± 0.05
	*TTR*	Transthyretin	−2.03 ± 0.08

Mean hepatic gene expression and SEM of the scrapie group at 23 wpi with fold change ≥1.5 relative to the mean expression in the control group scaled to 1

### Gene expression analysis in brain at 23 wpi

Gene expression differences in brain tissue are listed in Table 3. Out of 12 target genes analysed after pre-processing, only four were expressed with fold change ≥1.5. *Hp* and *Cp* were upregulated, and *ALB* and *TTR* were downregulated.

**Table 3:** Fold change of selected genes in brain tissue

Gene function	Gene Name		Fold Change ± SEM
Acute Phase Protein	*Hp*	Haptoglobin	7.91 ± 2.39
	*Cp*	Ceruloplasmin	1.67 ± 0.73
	*ALB*	Albumin	−1.64 ± 0.06
	*TTR*	Transthyretrin	−1.61 ± 0.17

Mean fold change and SEM in brain tissue in the scrapie infected group at 23 wpi relative to the expression in the control group

## Discussion

Classical scrapie is known to present with a variety of clinical signs, and clinical presentation is dependent on *PRNP* genotype, PrP^Sc^ strain, infective dose and age of host at infection. Our experimental model resembles natural infection and illustrates the “worst-case” scenario which can occur naturally when the right conditions are present. Clinical signs of disease in the scrapie group was first observed at 19 wpi. The symptoms aggravated quickly, and by 23 wpi, two lambs had reached the terminal stages. The very short incubation period was dominated by sudden progressive weakness, followed by rapid worsening and necessitating euthanasia at an early age. Hence, the observed clinical presentation is not typical for classical scrapie known to present in older animals, which is characterised by a slow and long incubation period with signs of progressive neurodegeneration associated with dissemination and accumulation of PrP^Sc^ within the CNS. Although this experimental scrapie model attempts to eliminate many of the factors that generate disease variation, individual variation still occurred. Individual progression of clinical disease and expression profiles in blood and tissues differs between animals. The APR is fast, and the level of inflammatory mediators, such as cytokines, changes continuously. Cytokines are multifunctional (both pro-inflammatory and anti-inflammatory) intra-cellular and inter-cellular signalling molecules, operating in a very complex signalling network to initiate and fine-tune the immune response [34–38]. Their levels are tightly controlled within a narrow range by inhibitory mechanisms and antagonists, whilst APPs can increase quite dramatically depending on type of protein [39, 40].

In the present study, we have investigated the expression of a selection of genes in blood approximately half-way through the incubation period, at 12–16 wpi, and at the clinical end-stage, at 22–23 wpi. Expression levels of selected genes were also investigated in liver and brain tissues, harvested at post mortem examination. In summary, the mean expression of six genes were significantly altered in the scrapie group compared to the control group. We were not able to calculate statistical significance in mean expression levels in brain and liver tissues, but we believe that a difference in mean expression levels greater than 1.5 fold change is of biological relevance and will be discussed. Despite our best efforts, only four genes in the brain tissue were differently expressed in the scrapie group, compared to the control group. These findings were unexpected as all the animals in the scrapie group were clinically affected and were positive for PrP^Sc^ in the brain on both IHC examination and WB (Fig. 1).

**Fig. 2:**
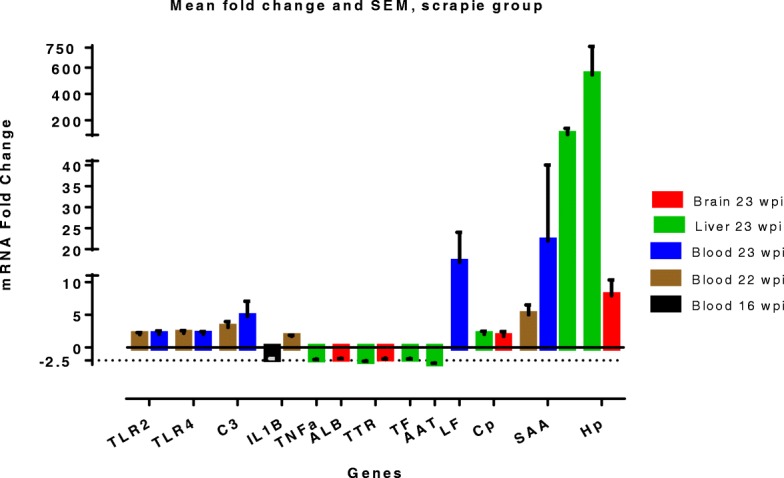
Bar diagram presenting average fold change and SEM in the scrapie group of selected genes. The fold change value is scaled, in each data set, to the mean fold change value of the control group equals 1 (one)

Our overall results, presented in Fig. 2, indicate an innate immune response, where genes involved in pattern recognition, complement system and acute phase proteins, are significantly altered in blood at the clinical stages (22 and 23 wpi). Mean gene expression levels of brain and liver tissues in the scrapie group show ≥1.5 fold change of several APPs. These findings coincide well with our earlier reports on increased serum levels of APPs, and identification of SAA as being a discriminating protein by proteomic analysis of serum from the scrapie group [29, 41].

Prion disease and the innate immune system have previously been reviewed by Bradford and Mabbott, and activation of cells within the innate immune system is critical to peripheral and central prion pathogenesis [42]. Gene network analyses of brain tissue from prion infected mice show that increased expression of C3, PRRs (including TLR2), and other receptors involved with PrP^Sc^ recognition and uptake are one of the first transcriptome changes [43, 44]. TLR2 is one of the, at least, ten members of the ovine TLRs family which are membrane-bound PRRs [45]. Our results show significantly increased *TLR2* and *TLR4* expression in blood from 22 wpi, thus, indicating an increased transcription of these receptors in circulating blood cells. Both these TLRs are situated on the cell surface and recognize a variety of microbial components, and activation will trigger expression of several genes and signalling pathways involved in the immune response [37, 46, 47]. Activation of different TLRs leads to specific gene expression profile patterns, and the specific signalling pathways in prion diseases are not yet fully revealed. TLR2 and TLR4 signalling pathways share a common MyD88-dependent pathway which results in production of inflammatory cytokines. In addition, TLR4 stimulation can also initiate a MyD88-independent pathway [46]. Prinz et al. demonstrated that MyD88^−/−^ mice were highly susceptible to prion infection, and thus pathogenesis and neuroinvasion are not solely dependent on the MyD88-dependent pathway [48]. Spinner et al. showed that scrapie pathogenesis occurs more rapidly in mice mutant of TLR4 signalling and concludes that this may be due to a reduced innate response to PrP^Sc^ [49]. The TLRs may be involved in binding PrP^Sc^ and initiating the innate immune response [42, 48–52]. Alongside the TLRs, there is a significant increased expression of C3 in blood at both 22 and 23 wpi. Binding of PrP^Sc^ to complement components plays an important role in getting the agent to lymphoid tissue and early accumulation in FDCs [53]. PrP^Sc^ interacts directly with C1q and thereby activates C3 through the classical pathway [54–56]. The complement system, in general, sense and react quickly to danger signals and can potentially initiate a strong inflammatory response, which needs to be tightly balanced between activation and inhibition [54].

Binding to and activation of PRRs lead to the production of pro-inflammatory cytokines that function both protective and degenerative. Increased levels of pro-inflammatory cytokines are associated with the systemic inflammatory response seen in prion infection and other neurodegenerative diseases [57, 58]. Cytokine antibody array analysis of scrapie-infected Tg338 mice shows significant alteration in expression of interleukins in spleen, mesenteric lymph node, brain and serum at both preclinical and clinical stages of infection [59]. We analysed several different interleukins, but only the expression *IL1B* was significantly changed in blood in the scrapie group; first downregulated at 16 wpi and then upregulated at 22 wpi. IL-1 beta is a powerful pro inflammatory mediator, and increased levels initiate a negative feedback loop [35, 39].

Translation and release of proinflammatory cytokines initiate a wide range of systemic inflammatory effects, including the synthesis and release of APPs mainly from the liver, but also extrahepatically [36, 38, 47, 60]. The APPs are classified as positive or negative depending whether their serum concentration increases or decreases during the APR [36, 47, 61, 62]. Our results show changes at transcription levels of both negative and positive APPs at the end stage. *ALB*, *TTR* and *TF* are negative APPs, and these have decreased expression in brain and liver. *ALB* is the major negative APP and synthesis is down-regulated during the APR. Transthyretin is a transport protein in serum and CSF, and one of its functions is inhibition of interleukin-1 production by monocytes and endothelial cells, and a decrease may, thus be pro-inflammatory [36]. *TTR* expression is reduced during the APR and the protein has been seen to decrease in serum in late stage scrapie in sheep [63, 64].

Of the positive APPs, *LF*, *Cp*, *SAA* and *Hp* have increased expression at clinical end stage, while *AAT* was down regulated. Alpha-1 antitrypsin (AAT) is a circulating serine protease inhibitor (serpin) and an acute phase protease, and it is classified as a minor positive acute phase protein in ruminants [64]. It is synthesised and secreted from the liver, and it plays an important role in the control of the inflammatory response [47, 65]. Serpinopathies and AAT deficiency have been associated with protein misfolding diseases [65, 66]. We found a decreased transcription of *AAT* mRNA in the liver in the scrapie group compared to the control group.

Gene expression of lactoferrin (LF), another minor positive APP, was increased by a 13.7-fold in blood in the scrapie group at 23 wpi. LF is a multifunctional glycoprotein with great iron-binding affinity and it is produced by mucosal epithelial cells [67]. It is involved in regulation of iron absorption and immune responses, and it has been suggested that LF has multifunctional antiprion activities [68]. Neutrophils are the source of lactoferrin in serum [69].

Ceruloplasmin (Cp) is a moderate APP with important biological functions. It is the major copper-carrying protein in blood, it plays a role in iron homeostasis and has oxidase activity in the CNS [47, 70–72]. Cp is mainly produced in the liver, and we detected an increased transcription in liver tissue in the scrapie group at 23 wpi. This coincides well with our previous finding of increased serum levels of ceruloplasmin at 23 wpi [29].

Serum amyloid A (SAA) and haptoglobin (Hp) are major APPs in sheep and increased serum levels are associated with a number of diseases. These proteins are primarily synthesised by hepatocytes, but other tissues and cell types are able to produce these two locally, though at a much lower level [47]. Our results show an increased transcription of both genes in the scrapie group at the clinical end stage. *SAA* gene expression in blood was significantly increased at both 22 and 23 wpi, with an average fold change of 5.00 and 21.99, respectively. In liver tissue, the mean expression level was increased by an average 89.98 fold change in the scrapie group. These results indicate that the increased level of SAA in in serum, reported earlier, originates both from increased synthesis in the liver and in the blood.

*Hp* showed increased gene transcription in both liver and brain tissue, with a mean fold change of 542.86 and 7.91, respectively. This indicates an increased synthesis of Hp hepatically and extrahepatically within the brain in the scrapie group at 23 wpi.

There was considerable variation in relative expression of individual gene expression within the scrapie group, indicated by the SEM values (Tables 1-3). One animal stands out in gene expression fold change in liver and blood, and on clinical presentation (individual data not shown). We believe there is relationship between the magnitude of the systemic acute phase response and clinical presentation in these animals. The acute phase response leads to a number of behavioural, physiological, biochemical and nutritional changes [36]. The brain recognizes proinflammatory cytokines as molecular signals of sickness, and metabolic and behavioural changes are initiated. These changes are collectively called “sickness behaviour” [73]. These peripherally released cytokines communicate and act on the brain via a fast neural pathway through afferent neurons (especially the vagus nerve), and via a slow humoral pathway [37]. In this sense, the immune- and nervous systems are in close contact. The afferent branches of the vagus nerve are involved in transmitting signals of inflammation to the brain via cytokine receptors [37]. Dendritic cells and macrophages are present in close association to vagal nerve fibres and they are important in the immune-brain communication [74]. Dendritic cells and macrophages express membrane TLRs and can produce proinflammatory cytokines upon activation, and at the same time, these cell types play a role in peripheral prion pathogenesis by interacting with PrP^Sc^ [42, 75]. Immune signals and PrP^Sc^ neuroinvasion may share common neural pathway transmitting immune messages and PrP^Sc^ infectivity, respectively, from periphery to the brain, where DCs have an important role [37, 76]. The innate immune system and the nervous system are, thus closely linked through common hormonal and neuronal routes. The ANS and CNS enhance and dampen the inflammatory response, and regulation through negative feedback loops are important. Any interference may have deleterious consequences. Recently, Salvesen et al. suggested that PrP^C^ is one of the modulators of the innate immune response, and loss of PrP^C^ prolonged sickness behaviour and stimulated pro-inflammatory activity [77].

Similar experimental scrapie models in sheep show PrP^Sc^ accumulation in peripheral lymphatic tissue and gene expression changes about half-way through the incubation period, while the animals appear asymptomatic [20, 22, 24, 43]. We believe the situation in the scrapie group was similar, an asymptomatic incubation period was followed be a short clinical end stage characterised by progressive apathy, dirty wool and passiveness. By 23 wpi two sheep were recumbent, weak, incapacitated and responded poorly to external stimuli. Sheep are very good at hiding behaviour changes as indicators of disease, thus presence of clinical signs cannot always be relied upon [78]. Sickness behaviour can be both beneficial and disadvantageous to the animal, and it can, to some extent, be overruled by fear and mothering behaviour [73, 79]. Why the long asymptomatic incubation period, despite the ongoing accumulation of PrP^Sc^ in LRS and CNS and gene expressions alterations, is not fully understood. Many of the genes involved are part of the innate immune system, and it is known that a prolonged and excessive inflammatory response can have negative or even lethal effects [34, 37, 80].

The particular disease phenotype seen in present study, is the result of the interaction between donor material, dosage and time of inoculation and recipient genotype, also described by González et al. [81]. The inoculation at birth before access to colostrum results in an effective and fast uptake of PrP^Sc^ across the susceptible new-born gut, similarly described by Tabouret et al. [24, 28]. These lambs have a characteristic dissemination of PrP^Sc^ throughout peripheral lymphatic tissues well in advance of PrP^Sc^ detection in the brain. The early and progressive development of clinical signs is unusual and have only been described earlier in a few rare cases of natural scrapie by Ulvund, although not at such a young age [82]. The histopathological examinations of the brain from these pre-colostrum inoculated lambs revealed only moderate cytopathological changes, with a mild to moderate gliosis with few vacuolated neurons. This is in somewhat in contrast to an earlier experimental model, described by Ersdal et al., where lambs of the same genotype (VRQ) were orally inoculated with brain tissue homogenate at ages between 46 and 61 days of age (mean 55.6 days).

## Conclusion

We believe our experimental model produces a “worst-case” scenario which can occur in natural scrapie settings. Homozygous VRQ offspring from subclinically infected ewes, can become infected at birth and develop clinical scrapie similar to the cases described here. In naturally occurring scrapie, such animals will pose a threat to other susceptible sheep through environmental contamination due to high infectivity. The young age and atypical clinical presentation of these cases can result in misdiagnosis and not be recognisable as scrapie. Further work is needed to investigate the connection between the innate immune system and prion diseases, and whether the severe response we observed in our model can be linked to loss of modulatory function of PrP^C^.

## Additional files

Additional file 1: Quantity and quality calculations of extracted RNA. Descriptive statistical presentation of quantitative and qualitative properties of the extracted RNA from blood, liver tissue and brain tissue. (DOCX 13 kb)

Additional file 2: Primers and reaction conditions. Gene functional class, gene abbreviation, gene name, gene bank entry and primer sequences used in the real time qPCR analysis. Calculated PCR efficiency and correlation are also listed. (XLSX 28 kb)

## Abbreviations

AAT: Alfa-1 antitrypsin
ALB: albumin
ANS: Autonomic nervous system
APP: Acute Phase Protein
APR: acute phase response
C3: complement component 3
cDNA: complementary DNA
CNS: Central nervous system
Cp: Ceruloplasmin
CSF: cerebrospinal fluid
DCs: Dendritic cells
DNA: Deoxyribonucleic acid
FC: fold change
FDCs: follicular dendritic cells
Hp: Haptoglobin
IHC: immunohistochemistry
IL1B: Interleukin-1-beta
LF: Lactoferrin
LRS: Lymphoreticular system
mRNA: Messenger ribonucleic acid
NTC: No template control
PNS: Peripheral nervous system
PRNP: Prion protein gene
PrP^**C**^: Normal cellular prion protein
PrP^Sc^: Abnormal isoform of prion protein
PRRs: pattern recognition receptors
RIN: RNA integrity number
RNA: Ribonucleic acid
RT-qPCR: Reverse transcription quantitative real-time polymerase chain reaction
SAA: Serum amyloid A
SEM: Standard errors of mean
TF: Transferrin
TLR2: Toll-like receptor 2
TLR4: Toll-like receptor 4
TLRs: Toll-like receptors
TNFa: Tumor necrosis factor alfa
TSE: Transmissible spongiform encephalopathy
TTR: Transthyretin
WB: western blot
wpi: weeks post inoculation
