# Supplementary material for: Expression of selected genes isolated from whole blood, liver and obex in lambs with experimental classical scrapie and healthy controls, showing a systemic innate immune response at the clinical end-stage
Source: BMC Vet Res. 2018 Sep 12;14:281. doi: 10.1186/s12917-018-1607-9 (PMC6134718; doi:10.1186/s12917-018-1607-9)
Supplement: Supplementary file 1 — Quantity and quality calculations of extracted RNA. Descriptive statistical presentation of quantitative and qualitative properties of the extracted RNA from blood, liver tissue and brain tissue. (DOCX 13 kb) [file 12917_2018_1607_MOESM1_ESM.docx]

**Additional file 1 - Quantity and quality calculations of extracted RNA**

|  | blood | | liver | | brain | |
| --- | --- | --- | --- | --- | --- | --- |
|  | ng/µl | RIN | ng/µl | RIN | ng/µl | RIN |
| Mean | 53.8 | 8.7 | 572.3 | 7.9 | 160.4 | 6.7 |
| Standard Error | 2.3 | 0 | 52.2 | 0.1 | 15.5 | 0.1 |
| Median | 51.6 | 8.7 | 647 | 7.8 | 157 | 6.7 |
| Standard Deviation | 23.2 | 0.4 | 255.6 | 0.6 | 46.5 | 0.2 |
| Range | 130.9 | 2.1 | 963 | 2.1 | 144 | 0.6 |
| Minimum | 14.9 | 7.5 | 31 | 7.1 | 82 | 6.4 |
| Maximum | 145.8 | 9.6 | 994 | 9.2 | 226 | 7 |
| Count | 98 | 98 | 24 | 24 | 9 | 9 |

Descriptive statistical presentation of quantitative and qualitative properties of the extracted RNA from blood, liver tissue and brain tissue.
